# A Defined-Area Bonding Approach for Microtensile Testing: A Reliable Alternative to Monoblock Sectioning for High-Hardness Restorative Materials

**DOI:** 10.3390/jfb17030141

**Published:** 2026-03-11

**Authors:** Koji Yamashita, Chiharu Kawamoto, Yu Toida, Shimpei Kawano, Shuhei Hoshika, Hidehiko Sano, Atsushi Tomokiyo

**Affiliations:** 1 Department of Restorative Dentistry, Graduate School of Dental Medicine, Hokkaido University, Kita 13 Nishi 7, Kita-ku, Sapporo 060-8586, Japan; rinnum@den.hokudai.ac.jp (Y.T.); hoshika@den.hokudai.ac.jp (S.H.); 2 Department of Restorative Dentistry, Faculty of Dental Medicine, Hokkaido University, Kita 13 Nishi 7, Kita-ku, Sapporo 060-8586, Japansano@den.hokudai.ac.jp (H.S.); tomokiyo@den.hokudai.ac.jp (A.T.); 3 Sapporo Prison, Sapporo Regional Correction Headquarters, Correction Bureau, Ministry of Justice, Government of Japan, 1-5-1 Higashinaebo 2-jo, Higashi-ku, Sapporo 007-0802, Japan

**Keywords:** new methodological approach, microtensile bond strength, monoblock sectioning technique, defined-area bonding approach, resin cement

## Abstract

Background: The microtensile bond strength (μTBS) test is the gold standard for evaluating adhesive performance in restorative dentistry. However, the conventional non-trimming technique—referred to in this study as the monoblock sectioning technique (MST)—is difficult to apply to hard and brittle CAD/CAM materials such as zirconia and ceramics, thereby limiting test reproducibility. This study compared a newly developed defined-area bonding (DAB) method with MST to determine whether DAB could serve as a reliable specimen preparation technique for μTBS testing. Methods: CAD/CAM resin blocks and resin core materials were bonded using either ESTECEM II or Panavia V5. MST specimens were obtained by bonding the blocks first and subsequently sectioning them into individual beams. In contrast, DAB specimens were produced by pre-shaping the sticks and bonding them within a defined 1 mm^2^ area. μTBS, failure modes, and fracture/interface morphology (SEM) were evaluated. Results: MST produced significantly higher μTBS values than DAB (*p* < 0.001), with central MST beams showing the highest bond strengths. DAB values were statistically equivalent to MST peripheral values for both cements. More than 80% of failures were cohesive within resin cement across all groups. SEM revealed uniform cement layer thickness (50–60 μm) and similar peripheral-like fracture patterns in DAB specimens. Conclusions: Although MST yielded higher μTBS overall, the DAB method produced bond strengths equivalent to the MST peripheral region and demonstrated consistent fracture characteristics. Because DAB requires minimal cutting, it offers a promising, reproducible approach for μTBS testing of high-hardness materials that are otherwise difficult to section.

## 1. Introduction

Since the late 1970s, the introduction of adhesive resin composite restorations has driven continuous demand for improved bonding systems [1]. Traditionally, bond strength was assessed in tensile or shear modes using a universal testing machine (e.g., Instron), with one measurement obtained per tooth. However, with the advent of adhesive primers [2,3], conventional methods showed improvement in bond strength, eventually plateauing around 18–20 MPa [4]. Tensile bond strength is known to depend on the bonding area [5]. Studies have reported that measuring bond strength over larger surface areas leads to a higher incidence of cohesive failure in dentin [6]. Moreover, when uniform materials undergo tensile testing, tensile strength tends to decrease with increasing specimen size [7]. This suggests that larger specimens likely contain more defects than smaller ones, making it challenging to identify significant differences in bond strength using conventional shear or tensile tests.

To overcome these limitations, the microtensile bond strength (μTBS) test was introduced in 1994 [5]. This method demonstrated that using a small bonding area could yield higher bond strengths [8]. Furthermore, the μTBS test more frequently results in adhesive failures at the bonding interface and has proven to be more discriminative than traditional macro-shear tests [4,6,9,10,11,12,13]. A significant advantage of the μTBS test is its ability to measure tensile strength at different locations within a single tooth [5], allowing multiple data points to be collected from one specimen, which is beneficial for sample acquisition. These benefits have led to its widespread adoption by numerous research institutions, establishing it as one of the most standard and versatile bond strength testing methods today.

Initially, the μTBS test employed hourglass-shaped specimens trimmed to a rectangular cross-section (the trimming technique) [5]. Subsequently, bar-shaped specimens with a 1 mm × 1 mm cross-sectional area (the non-trimming technique) were developed [14]. Reports indicated that bond strengths obtained with this method were comparable to those from the trimming technique [14]. These findings suggest that non-trimmed specimens are simpler to fabricate and less susceptible to operator technique. Consequently, recent research predominantly utilizes the non-trimming technique.

Recent advancements in bonding systems, alongside improvements in processing technologies such as CAD/CAM systems and increasing patient demand for esthetics, have facilitated the use of tooth-colored restorative materials such as resin, zirconia, porcelain, and lithium disilicate [15,16,17]. However, specimen preparation using the non-trimming technique requires sectioning with a low-speed diamond saw (e.g., ISOMET 1000), which poses significant difficulties when applied to hard and brittle materials such as zirconia, porcelain, and lithium disilicate [18,19,20,21]. Their high hardness and susceptibility to chipping during cutting processes present challenges in obtaining standardized specimens, thereby limiting the reliability and reproducibility of bond strength measurements.

In Japan, CAD/CAM resin blocks are widely used in insurance-covered restorative treatments and have become a practical choice for inlays and crowns. Their high material uniformity, achieved through CAD/CAM processing, makes them particularly suitable as substrates for bond strength testing, since variability from natural tooth anatomy can be avoided [22,23]. While these blocks can be sectioned with conventional cutting machines, their ease of handling contrasts with the challenges posed by harder or more brittle materials such as zirconia or porcelain. This study therefore employed CAD/CAM resin blocks and resin cores as standardized substrates to minimize variability and ensure reproducibility, while also aiming to evaluate a new preparation method that could be extended to materials that are difficult to cut or prone to fracture.

In this study, we compared the conventional monoblock sectioning technique (MST) with a newly proposed defined-area bonding (DAB) method. While MST involves bonding block-shaped substrates and then cutting them to create multiple sticks, DAB involves bonding pre-machined substrates together within a defined area. Because the DAB method requires little cutting, it may be applicable to hard materials such as zirconia and ceramics, making it a promising option for μTBS testing of high-hardness materials [24]. Based on the above, the null hypothesis in this study was that the performance of μTBS specimens prepared using the DAB method is no different from that prepared using the conventional MST method.

## 2. Materials and Methods

### 2.1. Materials

All materials used in this study are listed in Table 1. ESTECORE (Tokuyama Dental, Tokyo, Japan) was selected as the resin core material, and ESTELITE P Block (Tokuyama Dental, Tokyo, Japan) was used as the CAD/CAM resin block substrate. Two resin cements were evaluated: ESTECEM II (Tokuyama Dental, Tokyo, Japan) and Panavia V5 (Kuraray Noritake Dental, Tokyo, Japan). Surface treatments were applied following the manufacturers’ instructions.

For the DAB technique, a 0.08 mm thick PTFE tape (AS FLON Tape, AS ONE, Osaka, Japan) with a circular opening (1.13 mm diameter; ~1 mm^2^) was used to define the bonding area.

### 2.2. Preparation of Resin Core and Block Substrates

**Figure 1:**
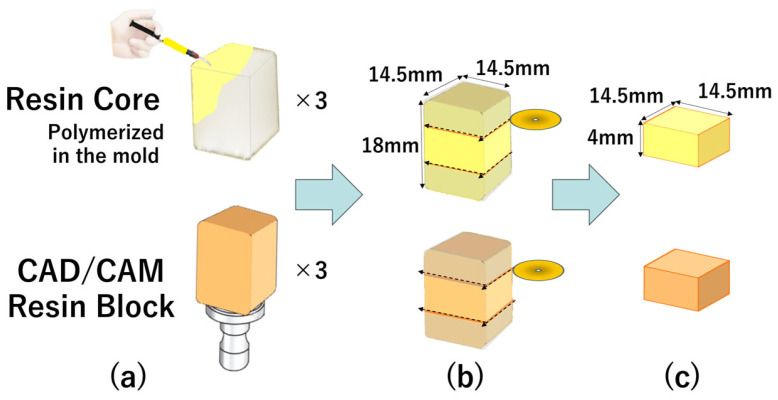
Sample preparation process. (**a**) A block of ESTECORE was prepared using a mold (14.5 mm × 14.5 mm × 18 mm). (**b**) The block was then sectioned into 14.5 mm × 14.5 mm × 4 mm slices using an Isomet 1000 (Buehler). (**c**) The cut surfaces were sandblasted with 50 µm alumina for 10 s, followed by ultrasonic cleaning for 2 min. The slices were then rinsed and dried.

Blocks of ESTECORE were fabricated using a stainless-steel mold (14.5 × 14.5 × 18 mm) and light-cured according to the manufacturer’s protocol. Each block was sectioned into 14.5 × 14.5 × 4 mm slices using a low-speed diamond saw (Isomet 1000, Buehler, IL, USA). The cut surfaces were sandblasted with 50 µm alumina for 10 s, ultrasonically cleaned for 2 min, rinsed, and air-dried. A schematic overview of the preparation procedure is shown in Figure 1.

### 2.3. Specimen Preparation Technique

**Figure 2:**
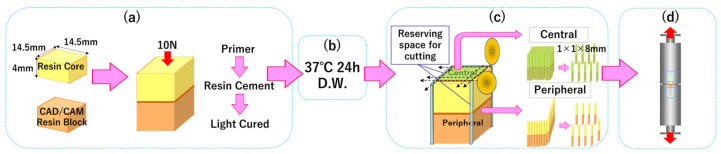
MST method. (**a**) CAD/CAM resin blocks and resin core blocks were bonded together using specific luting agents and protocols; (**b**) specimen storage; (**c**) preparation and classification of bonded specimen beams: the bonded specimens were prepared using a cutting machine and categorized into central and peripheral portions relative to the bonded interface; (**d**) μTBS Test: The μTBS of the beams was measured using a tensile testing machine.

**Figure 3:**
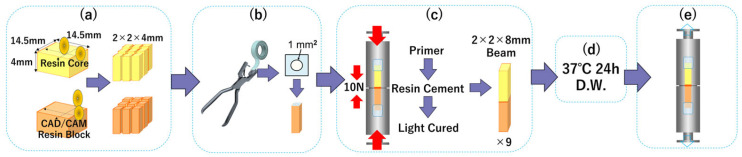
DAB technique. (**a**) Specimen preparation: stick-shaped specimens of both CAD/CAM resin and resin core were prepared using a cutting machine; (**b**) teflon tape application to the bonding surface of the CAD/CAM resin stick specimens; (**c**) bonding procedure; (**d**) storage of beams; (**e**) μTBS Test: the μTBS of the beams was subsequently measured using a tensile testing machine.

Two specimen preparation techniques were compared: the Monoblock Sectioning Technique (MST) and the Defined-Area Bonding (DAB) technique. The procedures for each method are summarized below. Figure 2 shows the process of MST technique and Figure 3 shows the process of DAB technique.

#### 2.3.1. MST (Monoblock Sectioning Technique)

##### Block Bonding

A CAD/CAM resin block and a resin core block were bonded using either ESTECEM II or Panavia V5.

ESTECEM II (MST): Bondmer Light-Less was applied to both substrates and air-dried. ESTECEM II was applied, the blocks were pressed together under a 10 N load, excess cement was removed, and light curing was performed from four directions (20 s per side) while maintaining the load for 3 min.

Panavia V5 (MST): The CAD/CAM block was etched with 35% phosphoric acid for 5 s, rinsed, and dried, then treated with Ceramic Primer Plus. The resin core block was treated with Tooth Primer for 20 s and gently air-dried. Panavia V5 was applied, followed by bonding under a 10 N load, removal of excess cement, and light curing for 20 s from each side while maintaining pressure for 3 min. All bonded blocks were stored in distilled water at 37 °C for 24 h.

##### Stick Fabrication and Classification

Bonded blocks were sectioned into stick-shaped specimens (1 × 1 × 8 mm). Beams were categorized based on their position relative to the bonded interface: Central–ESTECEM (MST), Peripheral–ESTECEM (MST), Central–Panavia (MST), Peripheral–Panavia (MST).

Each category contained 27 beams (9 beams × 3 blocks). Figure 2 shows the process of MST technique.

#### 2.3.2. DAB (Defined-Area Bonding Technique)

##### Preparation of Pre-Machined Sticks

Stick-shaped specimens (2 × 2 × 4 mm) were sectioned from CAD/CAM resin blocks and resin core blocks using the Isomet 1000. All bonding surfaces were sandblasted and cleaned following the same protocol described in Section 2.2.

##### Bonding Area Definition

A PTFE tape mask with a 1 mm^2^ circular opening was applied to the bonding surface of the CAD/CAM stick to create a defined bonding area.

##### Bonding Procedure

Bonding procedures for ESTECEM II and Panavia V5 followed manufacturers’ instructions, identical to those used for MST. Each pair of sticks was bonded under a 10 N load for 3 min using a tensile testing jig. Excess cement was removed prior to light curing.

The resulting bonded specimens measured 2 × 2 × 8 mm. All beams were stored in distilled water at 37 °C for 24 h.

##### Classification

DAB specimens were divided into two groups (n = 27 each): ESTECEM (DAB) and Panavia (DAB).

Figure 3 shows the process of DAB technique.

### 2.4. Microtensile Bond Strength (μTBS) Testing

μTBS testing was performed using a universal testing machine (EZ-Test, Shimadzu, Kyoto, Japan) at a crosshead speed of 1 mm/min. Each beam was aligned so that the tensile load was applied perpendicular to the bonding interface.

### 2.5. Sample Size Calculation

The required sample size for the equivalence analysis was estimated based on previously reported μTBS values for CAD/CAM resin-based materials, which typically show standard deviations of approximately 7–10 MPa. An equivalence margin of ±10 MPa was adopted for both resin cements, consistent with prior methodological studies evaluating μTBS preparation techniques. Using a two one-sided test (TOST) procedure with a one-sided α of 0.05 and a desired statistical power of 0.90, the minimum number of specimens required per group was calculated to be 27. This sample size ensures at least 90% power to detect equivalence when the true difference between DAB and MST-peripheral values falls within the predefined ±10 MPa margin. The final sample size used in the present study (n = 27 beams per group) met this requirement and therefore provided adequate power for the planned equivalence testing.

### 2.6. Statistical Analysis

Statistical analysis was conducted in three steps: (i) Overall effects of method (MST vs. DAB) and material (ESTECEM vs. Panavia): a two-way ANOVA was performed using block-level means (for MST) to compare test methods. Holm-adjusted post hoc contrasts were used. (ii) Regional differences within MST (center vs. peripheral): Paired *t*-tests were performed using beams obtained from the same block. (iii) Equivalence testing between DAB and MST peripheral: Exploratory equivalence testing using the Two One-Sided Tests (TOST) procedure was performed with an equivalence margin of ±10 MPa. A sample size of 27 beams per group provided 90% power.

### 2.7. Failure Mode Analysis

Failure patterns were examined using an optical microscope (Magnifier Light, AS ONE, Osaka, Japan). Failures were classified as follows: A: Adhesive failure, C: Cohesive failure within resin cement, M: Mixed failure.

### 2.8. SEM Analysis

#### 2.8.1. Fracture Surface Morphology

All fractured beams were initially examined under SEM to identify the characteristic morphological features of each group. Representative specimens were selected based on the consistency and recurrence of these features within each group. Although the examiner was aware of the group allocation, all specimens were examined prior to reviewing the corresponding μTBS results to minimize potential selection bias. Selected specimens were dried, sputter-coated with Pt–Pd, and examined under a scanning electron microscope (S-4800, Hitachi, Tokyo, Japan) at 10 kV.

#### 2.8.2. Bonding Interface Observation

Representative bonded interfaces were similarly prepared and examined to assess cement layer thickness and interfacial morphology.

## 3. Results

### 3.1. µTBS

**Figure 4:**
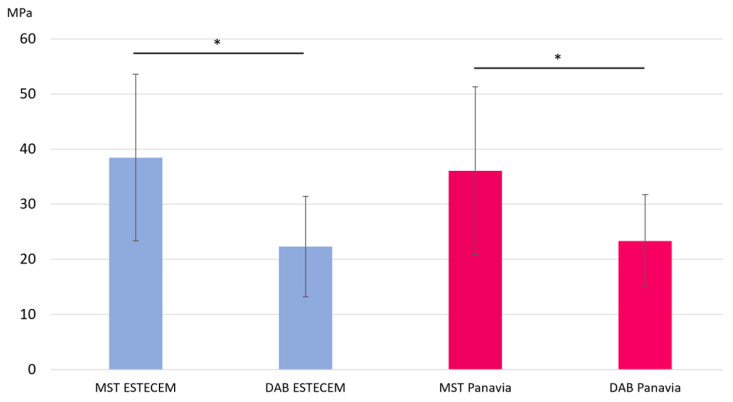
Bond Strength (MPa) for Each Group. Mean μTBS values (± SD) for MST and DAB across two resin cements (ESTECEM and Panavia). Error bars represent standard deviations. * Significant difference (*p* < 0.001).

The results of µTBS are shown in Figure 4, and the numerical values are summarized in Table 2. For MST, the ESTECEM group showed a mean bond strength of 38.46 ± 15.12 MPa, while the Panavia group showed 36.07 ± 15.22 MPa. In contrast, the DAB specimens exhibited lower values: 22.31 ± 9.12 MPa for ESTECEM and 23.33 ± 8.40 MPa for Panavia.

Two-way ANOVA revealed a significant main effect of preparation method (*p* < 0.001), with MST producing higher μTBS values than DAB, regardless of resin cement. Neither the main effect of material nor the interaction between method and material was statistically significant. Holm-adjusted contrasts confirmed significantly higher bond strength in MST for both ESTECEM and Panavia (*p* < 0.001 for both comparisons).

**Figure 5:**
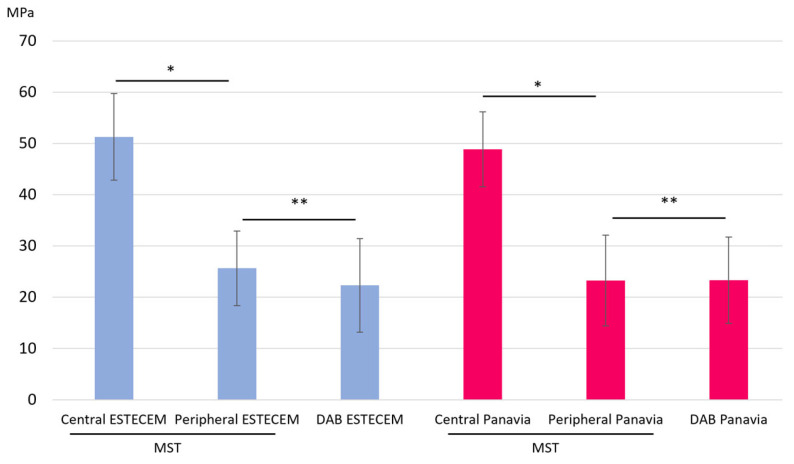
Bond Strength (MPa) for Each Group in central and peripheral regions. Dashed lines indicate the equivalence margin. * Significant difference between central and peripheral regions (*p* < 0.001). ** Statistical equivalence confirmed by TOST within the ±10 MPa margin.

When MST specimens were further classified into central and peripheral regions (Figure 5), central beams exhibited markedly higher μTBS than peripheral ones. The central ESTECEM group showed 51.28 ± 8.46 MPa, whereas the peripheral region showed 25.64 ± 7.29 MPa. Similarly, the central Panavia group showed 48.87 ± 7.31 MPa, compared with 23.27 ± 8.89 MPa in the peripheral region. Paired *t*-tests confirmed that central values were significantly higher than peripheral values for both materials (*p* < 0.001). The numerical values corresponding to Figure 5 are summarized in Table 3.

The DAB technique produced μTBS values comparable to those of the MST peripheral region. Equivalence testing using the TOST procedure supported statistical equivalence within the predefined ±10 MPa margin for both ESTECEM and Panavia groups.

### 3.2. Failure Mode Analysis

**Figure 6:**
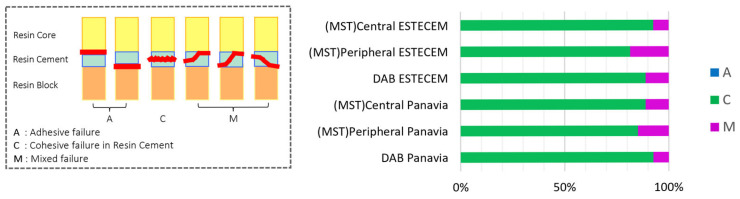
Classification of Failure Modes after Bonding Test. The position of the red line, which indicates the fracture area, enables classification of the failure modes into the following three types: A, adhesive failure; C, cohesive failure within the resin cement; and M, mixed failure. In all groups, cohesive failure within the resin cement accounted for more than 80%.

Figure 6 summarizes the failure mode distribution for all groups. Cohesive failure within the resin cement (type C) accounted for more than 80% of failures across all conditions. Adhesive and mixed failures (types A and M) occurred infrequently, with no notable differences between cement types or preparation techniques. The numerical distribution of failure modes is summarized in Table 4.

### 3.3. SEM Observation of Fracture Surfaces

**Figure 7:**
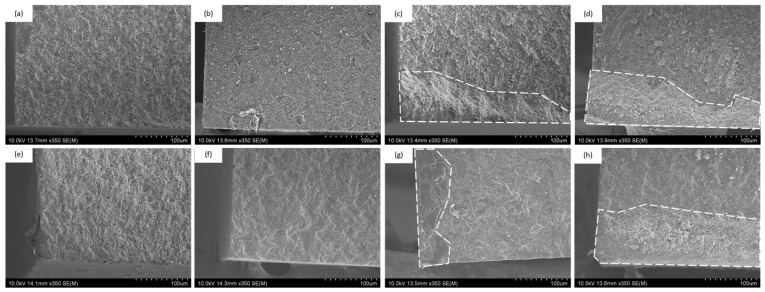
MST fracture surfaces: (**a**) Central ESTECEM resin core side, (**b**) Central ESTECEM resin block side, (**c**) Peripheral ESTECEM resin core side, (**d**) Peripheral ESTECEM resin block side, (**e**) Central Panavia resin core side, (**f**) Central Panavia resin block side, (**g**) Peripheral Panavia resin core side, (**h**) Peripheral Panavia resin block side.

Representative SEM images of fracture surfaces for MST specimens are shown in Figure 7. Central beams displayed relatively homogeneous and dense fracture patterns, whereas peripheral beams exhibited surface irregularities and morphological changes consistent with reduced polymerization quality at the margins.

**Figure 8:**
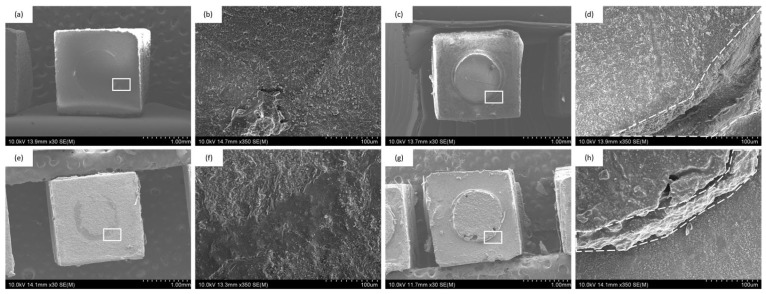
DAB fracture surfaces: (**a**) ESTECEM resin core side (×30); (**b**) Magnified SEM image of the surface shown in (**a**) (×350); (**c**) ESTECEM resin block side (×30); (**d**) Magnified SEM image of the surface shown in (**c**) (×350); (**e**) Panavia resin core side (×30); (**f**) Magnified SEM image of the surface shown in (**e**) (×350); (**g**) Panavia resin block side (×30); (**h**) Magnified SEM image of the surface shown in (**g**) (×350).

Figure 8 shows SEM images of DAB specimens. Their fracture surface morphology closely resembled that of the MST peripheral region, exhibiting similar microstructural features and surface texture.

### 3.4. SEM Observation of Bonding Interfaces

**Figure 9:**
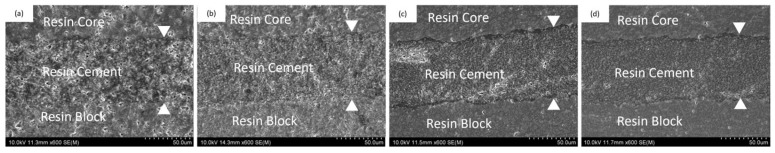
SEM images of bonding interfaces. (**a**) MST–ESTECEM, (**b**) MST–Panavia, (**c**) DAB–ESTECEM, (**d**) DAB–Panavia.

Figure 9 presents SEM images of the bonding interfaces for all groups. The resin cement layer thickness was consistently observed at approximately 50–60 μm, with no appreciable differences between MST and DAB specimens or between the two resin cements. The interfaces appeared continuous and free of defects.

## 4. Discussion

This study evaluated whether the newly developed DAB technique could provide μTBS results comparable to those obtained using the conventional MST method. The null hypothesis—that there would be no significant difference between the two specimen preparation techniques—was partially rejected because MST produced higher overall μTBS values. However, DAB exhibited bond strengths statistically equivalent to the MST peripheral region, indicating that the two methods are comparable under clinically representative polymerization conditions.

The significantly higher μTBS values in MST, particularly in the central region, are consistent with previous findings showing enhanced polymerization and more homogeneous cement curing at the center of bonded blocks [25,26,27,28,29,30,31,32]. The peripheral region, by contrast, is more strongly affected by the oxygen-inhibited superficial layer and by moisture-related interference during polymerization, both of which can reduce the degree of conversion and lead to lower bond strength [33,34,35,36]. Although this region receives higher light irradiance than the central portion, the stronger influence of oxygen inhibition at the free surfaces appears to be the dominant factor contributing to the reduced μTBS values observed at the periphery. This interpretation is consistent with classic findings showing that the maximum hardness of light-activated composites occurs not at the irradiated surface but 0.5–1.0 mm below it, with hardness decreasing toward both the surface and deeper regions [37]. In the present study, MST peripheral values were approximately half of the central values for both cements, aligning with established regional differences reported in previous microtensile-related studies [26,27]. The statistical comparison between the central and peripheral regions showed a highly significant difference, and the corresponding effect size indicated that the magnitude of this regional discrepancy was substantial. This reinforces the interpretation that the central region benefits from markedly reduced oxygen inhibition, resulting in a pronounced increase in μTBS.

The DAB method was conceived to predefine the bonding interface using CAD/CAM machining, enabling stick-shaped specimens to be fabricated prior to bonding and thereby avoiding post-bonding sectioning. Traditional specimen formats, such as 1 × 1 × 4 mm sticks or dumbbell-shaped pieces, often led to misalignment or excess cement accumulation. To overcome these drawbacks, a masking strategy was introduced using Teflon tape with a 1 mm circular aperture. Preliminary trials confirmed that ~80 μg of cement was sufficient to fill the aperture without overflow, and this condition was adopted in the present study. Resin cores were also selected as substrates to minimize variability associated with anatomical location or patient-specific differences, ensuring that the evaluation focused on the test method itself.

DAB specimens, by design, eliminate the central–peripheral discrepancy. Yet their bond strength values closely matched MST peripheral specimens rather than MST central ones. The non-significant *p*-value for the comparison between DAB and the MST peripheral region, together with the equivalence test results falling within the predefined ±10 MPa margin, indicates that the two methods are statistically and practically comparable. The small effect size further supports the conclusion that any differences between DAB and MST-peripheral values are negligible in magnitude. This suggests that the polymerization environment generated by the PTFE mask and the open bonding configuration of DAB resemble the periphery of MST blocks, where light exposure and oxygen inhibition can impede optimal polymerization. The SEM images support this interpretation, showing surface morphologies in DAB specimens similar to peripheral MST beams.

A major advantage of the DAB technique is its minimal need for sectioning. While MST is reliable for relatively soft materials such as CAD/CAM resin blocks, applying this method to brittle materials like zirconia, lithium disilicate, or porcelain often results in chipping, microcracks, or beam loss during sectioning [33]. These issues compromise the standardization of specimen dimensions and increase variability in μTBS results. In contrast, DAB allows pre-machined sticks to be fabricated without cutting the bonded interface, making it adaptable for high-hardness materials where MST is impractical or unreliable. Beyond these high-strength ceramics, the DAB approach may also be applicable to fragile materials such as glass ionomer cements, thereby broadening the range of substrates that can be evaluated with μTBS testing.

Cohesive failure within resin cement predominated (>80%) in all groups, indicating that the adhesive interfaces themselves were stronger than the cohesive strength of the cement. This finding reflects a well-established limitation of μTBS: cohesive failures can mask interfacial weaknesses and complicate interpretation of true adhesive performance [38,39,40]. Nevertheless, the similarity in failure modes across all groups demonstrates that neither MST nor DAB introduced artifacts that would alter fundamental adhesion processes.

The SEM examination of bonding interfaces showed consistent cement thickness (50–60 μm) and no major interfacial defects in any group. These results indicate that DAB does not compromise cement application or interfacial continuity despite the restricted bonding area imposed by the PTFE mask.

Given its equivalence to MST peripheral values and its ease of application to hard restorative materials, DAB can be considered a methodologically valid alternative to MST when sectioning is technically difficult or when specimen loss is expected. Moreover, the defined bonding area enhances reproducibility by eliminating variability caused by irregularly cut cross-sections—a common challenge in MST. However, further validation is required before the technique can be generalized to a broader range of restorative materials. In particular, brittle CAD/CAM ceramics such as zirconia and lithium disilicate represent important targets for future investigation, as these materials are highly susceptible to chipping and microcrack formation during MST sectioning. Because DAB eliminates post-bonding cutting, it may offer a distinct methodological advantage for such substrates, but this assumption must be experimentally confirmed.

In addition, the present study evaluated immediate bond strength without incorporating aging procedures. Recent studies have demonstrated that thermocycling can significantly reduce μTBS in indirect restorative materials, underscoring the importance of including aging protocols in bond-strength evaluation [32]. Thermocycling, long-term water storage, and other degradation protocols are essential for assessing the durability of adhesive interfaces, especially when polymerization conditions differ between techniques. Future studies should therefore examine whether DAB and MST exhibit comparable resistance to hydrolytic and thermal stresses, and whether the equivalence observed in the present study is maintained after aging. Such investigations will clarify the long-term reliability of DAB and determine its suitability for replacing MST in adhesion testing of next-generation restorative materials.

## 5. Conclusions

This study showed that while the conventional MST method produced higher overall μTBS values—especially in the central region—the DAB technique achieved bond strengths statistically equivalent to MST peripheral specimens. Cohesive failures predominated in all groups, and SEM analysis revealed comparable interfacial morphology and cement thickness. Because DAB minimizes cutting and avoids specimen damage common with brittle CAD/CAM materials, it provides a practical and reproducible alternative for μTBS testing when MST is difficult to perform.

## Figures and Tables

**Table 1:** Materials used in the study.

	Materials	Composition	Manufacturer	Lot
Resin Block	ESTELITE P BLOCK	Bis-MPEPP	Tokuyama Dental (Tokyo, Japan)	007069
		UDMA		
		NPGDMA		
		SiO_2_		
		Silica–Zirconia Filler		
		Pigments		
Resin Core	ESTECORE	Silica–Zirconia Filler		036061
		Bis-GMA		
		TEGDMA		
		Bis-MPEPP		
		Peroxide		
		Camphor quinone		
		Radical polymerization amplifier		
Cement	BONDMER LIGHTLESS	Acetone		036061
		Phosphoric acid monomer		
		Bis-GMA		
		TEGDMA		
		HEMA		
		MTU-6		
		Isopropyl alcohol		
		Water		
		Borate catalyst		
		Peroxide		
		γ-MPTS		
	ESTECEM II	Silica–Zirconia Filler		076081
		Bis-GMA		
		TEGDMA		
		Bis-MPEPP		
		Peroxide		
		Camphor quinone		
	K-Etchant Syringe	35% phosphoric acid	Kuraray Noritake Dental (Tokyo, Japan)	7N0118
		water		
		colloidal silica		
		dye		
	Clearfil Ceramic Primer Plus	3-trimethoxysilypropyl methacrylate		6Q0044
		10-MDP		
		ethanol		
	Panavia V5 Tooth Primer	10-MDP		6Q0067
		original multifunctional monomer		
		new polymerization catalysts		
		stabilizer		
		HEMA		
		water pH = 2.0		
	Panavia V5	Bis-GMA		AK0144
		TEGDMA		
		hydrophobic aromatic dimethacrylate		
		hydrophilic aliphatic dimethacrylate		
Tape	AS FLON Tapes	PTFE tape	AS ONE (Osaka, Japan)	―
		Si-PSA		

UDMA, urethane dimethacrylate; TEGDMA, triethylene glycol dimethacrylate; Bis-GMA, bisphenol A-glycidyl methacrylate; Bis-MPEPP, 2,2′-bis(4-methacryloxyate polyethoxyphenyl)propane; HEMA, 2-hydroxyethyl methacrylate; MTU-6, methacryloyloxydodecylpyridiniumbromide; γ-MPTS, gamma-methacryloxypropyltrimethoxysilane; 10-MDP, 10-methacryloyloxydecyl dihydrogen phosphate; NPGDMA, neopentylglycol dimethacrylate; PTFE, polytetrafluoroethylene; and Si-PSA, silicone pressure-sensitive adhesive.

**Table 2:** Numerical μTBS values (mean ± SD) corresponding to Figure 4 for MST and DAB specimens.

Test Method	Cement Type	Mean μTBS (MPa) ± SD
MST	ESTECEM	38.46 ± 15.12
DAB	ESTECEM	22.31 ± 9.12
MST	Panavia	36.07 ± 15.22
DAB	Panavia	23.33 ± 8.40

**Table 3:** Numerical μTBS values (mean ± SD) corresponding to Figure 5 for MST central, MST peripheral, and DAB specimens.

Test Method	Cement Type	Mean μTBS (MPa) ± SD
MST—Central	ESTECEM	51.28 ± 8.46
MST—Peripheral	ESTECEM	25.64 ± 7.29
DAB	ESTECEM	22.31 ± 9.12
MST—Central	Panavia	48.87 ± 7.31
MST—Peripheral	Panavia	23.27 ± 8.89
DAB	Panavia	23.33 ± 8.40

**Table 4:** Failure mode distribution (number of specimens and percentages) corresponding to Figure 6.

Group	A (n,%)	C (n,%)	M (n,%)
MST—Central ESTECEM	0 (0%)	25 (93%)	2 (7%)
MST—Peripheral ESTECEM	0 (0%)	22 (81%)	5 (19%)
DAB—ESTECEM	0 (0%)	24 (89%)	3 (11%)
MST—Central Panavia	0 (0%)	24 (89%)	3 (11%)
MST—Peripheral Panavia	0 (0%)	23 (85%)	4 (15%)
DAB—Panavia	0 (0%)	25 (93%)	2 (7%)

## Data Availability

The original contributions presented in the study are included in the article. Further inquiries can be directed to the corresponding author.
